# Experience and dissection device are more relevant than patient-related factors for operation time in laparoscopic sigmoid resection—a retrospective 8-year observational study

**DOI:** 10.1007/s00384-017-2896-3

**Published:** 2017-09-06

**Authors:** Dirk Weyhe, Verena Nicole Uslar, Navid Tabriz, Ina Burkowski, Ralf Heinzel, Andreas Müller, Annette Belling, Ferdinand Köckerling

**Affiliations:** 1 School of Medicine and Health Sciences, Clinic for Visceral Surgery, Pius-Hospital Oldenburg, Medical Campus University of Oldenburg, Georgstr. 12, 26121 Oldenburg, Germany; 2 Clinic for General and Visceral Surgery, Pius-Hospital Oldenburg, Oldenburg, Germany; 3 Clinic for General and Visceral Surgery, Hümmling Hospital, Sögel, Germany; 4 Department of Surgery and Center for Minimally Invasive Surgery, Academic Teaching Hospital of Charité Medical School, Vivantes Hospital, Berlin, Germany

**Keywords:** Laparoscopic surgery, Learning curve, Patient-specific factors, Patient safety, Thunderbeat

## Abstract

**Purpose:**

Surgical outcome is influenced by multiple patient-specific factors and operative expertise of the surgeon. Clinical relevance of medical technical innovations often remains unclear even though laparoscopic surgical procedures are characterized by continual advancement of various devices. Lately, in dissection and sealing technology, fast-cutting ultrasonic scissors are combined with simultaneous bipolar coagulation (bimodal dissection device (BDD)). We investigated how this new technology, operative expertise, and patient-specific factors (body mass index, age) influence operation time in laparoscopic-assisted sigmoid resection.

**Methods:**

Between 2008 and 2016, 161 laparoscopic sigmoid resections (52% conventional dissection device (CDD); 48% BDD) performed in a single center were retrospectively evaluated. Biometric patient data, complication rates, and surgery duration, reflecting the learning curve, were analyzed. Operations were performed by experienced surgeons (*n* = 3) and trainees (*n* = 4).

**Results:**

Minor postoperative complications (e.g., impaired wound healing, non-revisional secondary bleeding) occurred in 11 cases (6.8%). Major complications (e.g., bleeding requiring revision, anastomotic leakage) were observed in 3.7%. No heat-related coagulation damage was observed. BDD reduced operation time for both experienced (CDD 150 min, BDD 125 min; *p* < 0.001) and trainee surgeons (CDD 169 min, BDD 135 min; *p* = 0.036). Reduction of operation time (indicative of a learning curve in progress) was observed for all surgeons. The curve was steeper using BDD.

**Conclusions:**

Patient-specific factors did not have a significant effect on operation time. Even taking the learning curve into account, a combination of ultrasonic dissection and simultaneous bipolar coagulation reduces operation time of laparoscopic-assisted sigmoid resection, regardless of surgeon’s expertise.

## Introduction

The medical technical progress in laparoscopic surgery is driven, in particular, by enhanced visualization techniques such as 2D full HD, 3D full HD, and 4K as well as by increasingly more innovative dissection technologies [1–3]. Whereas in its infancy, laparoscopy involved the use of Röder loops, clip ligatures, and monopolar energy application for vascular occlusion, bipolar coagulation would later become established as the standard laparoscopic technique. A parallel development ushered in the use of high-frequency ultrasonic dissection which conferred the important advantage of local energy ablation without the risk of coagulation damage from transmitted energy flow as commonly reported for monopolar application [4, 5]. The drawback of high-frequency ultrasonic dissection derives from its poorer coagulation performance compared with monopolar and bipolar application. The dissection device Thunderbeat^™^ (bimodal dissection device (BDD)) generates brief start-up currents for effective bipolar coagulation, with simultaneous fast cutting speed using high-frequency ultrasound. In (pre)clinical trials, that combination was found to produce higher “burst pressure” of dissected arteries as well as a significantly faster tissue dissection time [3, 6–10]. However, these properties come hand in hand with markedly higher heat generation in the region of the scissor shanks. It is unclear to what extent these combined characteristics serve as an independent factor with a real impact on the overall operation time since multiple factors such as, e.g., the surgeon’s experience, patient’s body mass index (BMI), and local factors, additionally impact the operation time. The indicator operation chosen for the present retrospective analysis was laparoscopic sigmoid resection for symptomatic sigmoid diverticulitis or adenocarcinoma of the sigmoid colon.

Laparoscopic-assisted sigmoid resection has been used over the past 20 years for surgical treatment of sigmoid diverticulitis [11, 12]. Since then, this guideline-based, minimally invasive surgical procedure has become established as the method of choice [13–16]. Compared with open operations, it is associated with less postoperative pain, shorter hospital stay, and fewer long-term complications [17].

Thanks to the accretion of experience and the use of standardized surgical techniques, today laparoscopic intestinal resection is also being carried out in specialist oncology clinics, with comparably good, and in some cases even better, surgical outcomes and survival prospects [18]. The main benefit of minimally invasive surgical techniques resides in the shorter convalescence and associated shorter hospital stay [19]. Besides, there is less blood loss even while obtaining biopsy specimens of similar or superior histopathology quality [20–22]. On the other hand, the operation time for laparoscopic-assisted operations is reported to be longer [17, 23]. The latter appears to be attributable, among other things, to the fact that for minimally invasive procedures, the operation time depends on the surgeon’s experience and associated learning curve [24–28]. Moreover, the dissection device used can potentially have implications for the operation time. For example, manipulation of certain dissection devices requiring intermittent exchange of bipolar clamps and ultrasonic scissors is definitely more time consuming.

The transition from conventional dissection device (CDD) to BDD in our clinic in 2012 was mostly due to subjective assessment of all surgeons involved in the decision making. The present retrospective analysis therefore aimed to objectively determine to what extent this newly developed device constituted an independent factor with regard to the operation time, while taking the operative learning curve into account, and it evaluates to what extent there was a likelihood of specific complications, such as coagulation damage resulting from high heat generation or of secondary bleeding due to an inadequate sealing effect. This seems especially important, since, to date, little research has been carried out on the potential impact of medical technical devices on the surgical outcome because of the multifactorial variables involved. It is hardly conceivable that a single factor—in this case a dissection instrument—could have a greater impact on the operation time than factors such as operative expertise, intraoperative site, or patient-specific characteristics.

## Materials and methods

### Study design

**Fig. 1:**
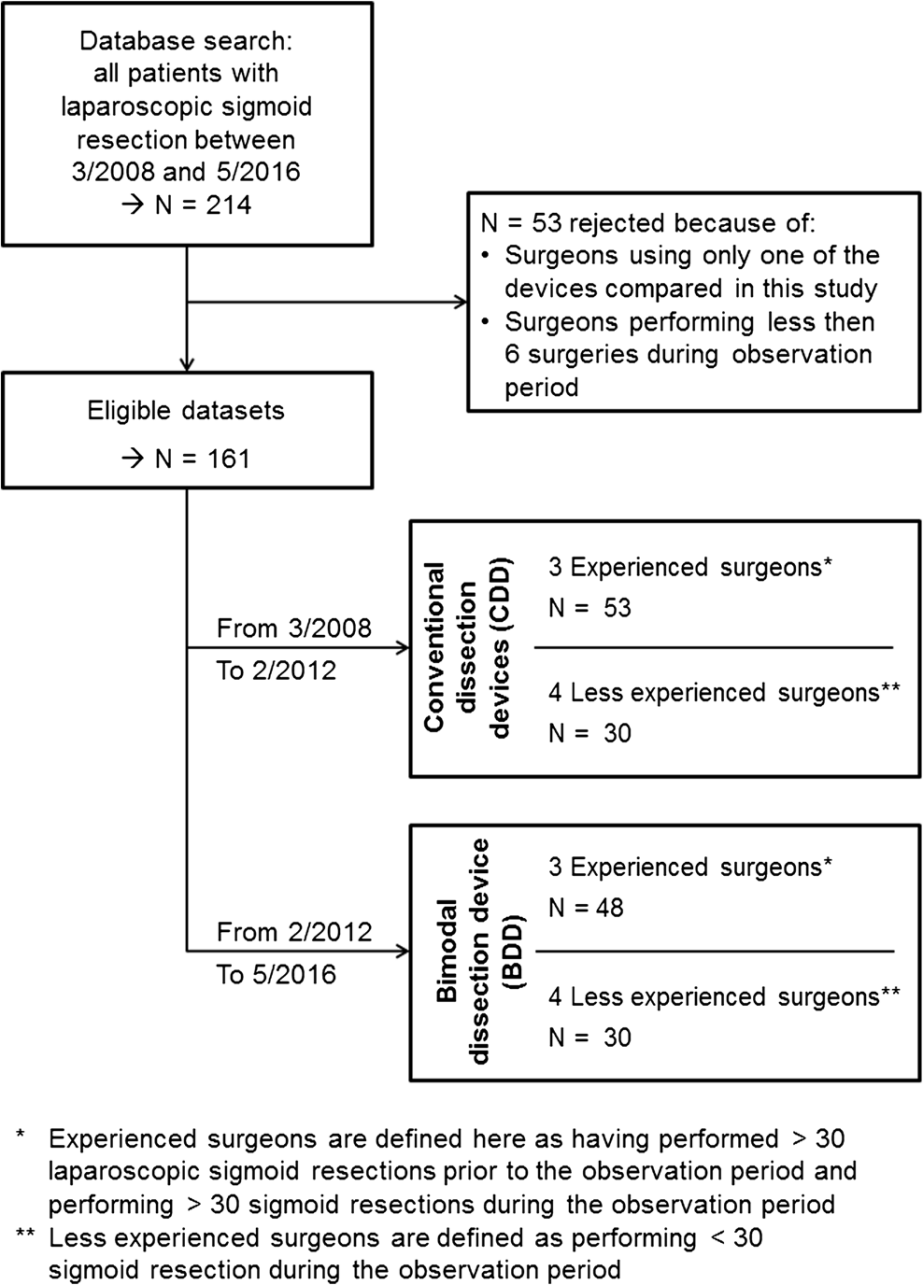
Flowchart of the study design

The observation period comprised 8 years (2008–2016). Between March 2008 and February 2012, bipolar clamps (Aesculap) in combination with ultrasonic dissector Harmonic ACE 36E (Ethicon) or bipolar dissection device LigaSure Blunt Tip LF 1637 (Covidien) were used. This combination of bipolar clamps with one of the aforementioned devices was subsumed as CDD. The instruments were selected by the respective surgeon, while the surgical technique had been set out in standard operating procedures (SOPs) as part of the continuing training program. In March 2015, all surgeons switched to using only Thunderbeat (BDD) as a replacement for the CDDs used prior to that (see also Fig. 1).

All laparoscopic-assisted sigmoid resections carried out in the Department of General and Visceral Surgery of the Pius Hospital Oldenburg were retrospectively evaluated. During the observation period (*n* = 214), laparoscopic sigmoid resections had been performed. The indication for elective surgical treatment was based on lack of response to conservative treatment or on diagnosis of complicated diverticulitis (as from types IIa and IIb as per the diverticular disease classification system). Furthermore, laparoscopic surgery was employed to treat all primary adenocarcinomas of the sigmoid colon (stage < c-T4). Operation data on the dissection and suturing time, biometric patient data (age and BMI), and ultrasonic device used (BDD vs. CDD) were recorded and evaluated while using anonymized surgeon codes.

### Surgeons

The operations were performed by *n* = 7 surgeons with different levels of experience in laparoscopic surgery since operations carried out by trainee surgeons were also included in the analysis. The surgeons were assigned to two different categories: group A = inexperienced (<30 laparoscopic sigmoid resections during the observation period) and group B = experienced (>30 laparoscopic sigmoid operations prior to the observation period). Furthermore, the experienced surgeons conducted more than 30 additional laparoscopic sigmoid resections during the observation period.

Excluded from the analysis were surgeons who had not performed the statistically prescribed minimum number of at least *n* = 3 procedures per dissection device during the observation period. Hence, *n* = 53 operations were not taken into account (Fig. 1). In total, *n* = 161 operations by *n* = 7 surgeons (*n* = 3 experienced, *n* = 4 trainee) were included in the analysis.

### Conduct of laparoscopic-assisted sigmoid resection

The operation is performed by all surgeons in accordance with the pertinent guidelines and internal standard procedures of the respective institution, with the patient in the lithotomy position. Laparoscopic preparation is effected while preserving the inferior mesenteric artery and protecting as far as possible the superior rectal artery in non-oncology resection settings. In oncology surgery, the mesenteric artery is excised around 1.5 cm above the origin of the aorta to protect the autonomic nervous plexus, and then, proximal mesorectal excision (PME) of the proximal rectum is performed. The intestinal tube is excised in the region of the proximal rectum, thus including excision of the “high-pressure zone” at the rectosigmoid junction. The left flexure is mobilized in the majority of cases to achieve tension-free anastomosis. Colorectal anastomosis was effected in all 161 cases by means of transanal stapler anastomosis (CEEA 31 mm, Covidien). Anastomotic control was performed by rectoscopy, air insufflations, and underwater tests. Patient postoperative mobilization was carried already on the day of operation in accordance with the fast-track concept, and forced transition to a normal diet was initiated on postoperative day 1.

### Statistics and figures

The programs IBM SPSS Statistics 24 or R 3.2.1 were used for all analyses. All analyses that included operation time as a variable were performed with non-parametric test methods since the Shapiro-Wilk test for standard distribution had revealed that the data did not follow the normal distribution. Therefore, the Mann-Whitney rank sum test was used for testing for significant differences (when comparing the frequency distribution of the operation time for CDD vs. BDD, for testing for differences in the operation time between BDD and CDD as well as differences between experienced and inexperienced surgeons and the subgroups BDD vs. CDD for experienced and inexperienced surgeons). The *t* test was used to test for significant differences between genders (i.e., BMI) or between BDD and CDD group in case of continuous variables with normal distribution (i.e., age and BMI). The chi-squared test was used for testing for differences between the BDD and CDD group in case of categorical variables (i.e., gender distribution, proportion of experienced and less experienced surgeons, number of oncological surgeries performed, and the number of complications). The Spearman rank test was used for testing for significant correlations between operation time, BMI, and age. In addition a multiple linear backward stepwise regression was performed to analyze which factor (i.e., type of diagnosis, type of dissection device used, BMI, age, and experience of the surgeon) primarily influences operation time. For this test, all operation time data was logarithmized to reduce positive skew of the data, and to thus obtain a data set with normal distribution.

For each statistical test, threshold for significance was *p* ≤ 0.05. All figures were created using Origin 2016G.

## Results

### Patients

In total, *n* = 161 patients who had undergone laparoscopic sigmoid resection because of diverticulitis or adenocarcinoma were analyzed. Of these, *n* = 82 (52%) were operated on using CDD and *n* = 79 (48%) using BDD (Table 1).

**Table 1:** Number of surgeries and surgery duration (median and range) stratified by dissection device (Harmonic C5 and LigaSure are later subsumed as CDD) and type of surgery (oncologic vs. non-oncologic resection)

	Harmonic		LigaSure		BDD	
	*N*	Duration (min)	*N*	Duration (min)	*N*	Duration (min)
Oncologic	2	181 (136 and 225)	0		20	136 (79–225)
Non-oncologic	73	150 (70–236)	7	148 (124–182)	59	136 (100–225)

The gender distribution was 64% (*n* = 103 female) vs. 36% (*n* = 60 male). The mean BMI was 26.9 ± 4.6 (female 26.2 ± 5, male 28.1 ± 3.7; *p* = 0.003), thus reflecting the WHO preobesity distribution patterns. The mean age was 60.8 ± 13.1 years.

To exclude further multifactorial influences on the operation time, the BDD and CDD patient groups were investigated for any differences in gender, age, and BMI. Significant differences between the BDD and CDD groups were detected only in the gender distribution, and in the distribution of oncological resections (see Table 1). Likewise, there was no significant difference between the BDD and CDD groups with regard to major complications such as, e.g., anastomotic leakage. All anastomotic leakages occurred after non-oncologic resection. There was no significant correlation between the operation time and the patient-specific variables age and BMI across the entire patient range or in the BDD and CDD subgroups (Spearman’s rank correlation; *p* always >0.1) (Table 2).

**Table 2:** Summary of descriptive statistics for eligible data sets

	BDD	CDD	*p* value
Number of operations	79	82	
Female	43 (54%)	60 (73%)	*p* = 0.009^a^
Oncological resections	20 (26%)	2 (2%)	*p* << 0.001^a^
Experienced surgeons	48 (61%)	53 (64%)	*p* > 0.05^a^
Anastomotic leaks	5 (6%)	1 (1%)	*p* > 0.05^a^
Mean age (years)	60.7 ± 13.2	60.9 ± 12.9	*p* > 0.05^b^
Mean BMI (kg/m^2^)	26.9 ± 4.5	26.8 ± 4.8	*p* > 0.05^b^

Given are either the number of patients (including the percentage) or the mean and standard deviation

^a^Chi-squared test

^b^ *t* test

### Surgeons

At the start of the observation period, the *n* = 3 experienced surgeons had an average of *n* = 12.6 (range 10–18) years of professional experience. The *n* = 4 trainee surgical assistants had <6 years of professional experience. On average, 20 laparoscopic sigmoid resections were performed per year during the observation period.

**Fig. 2:**
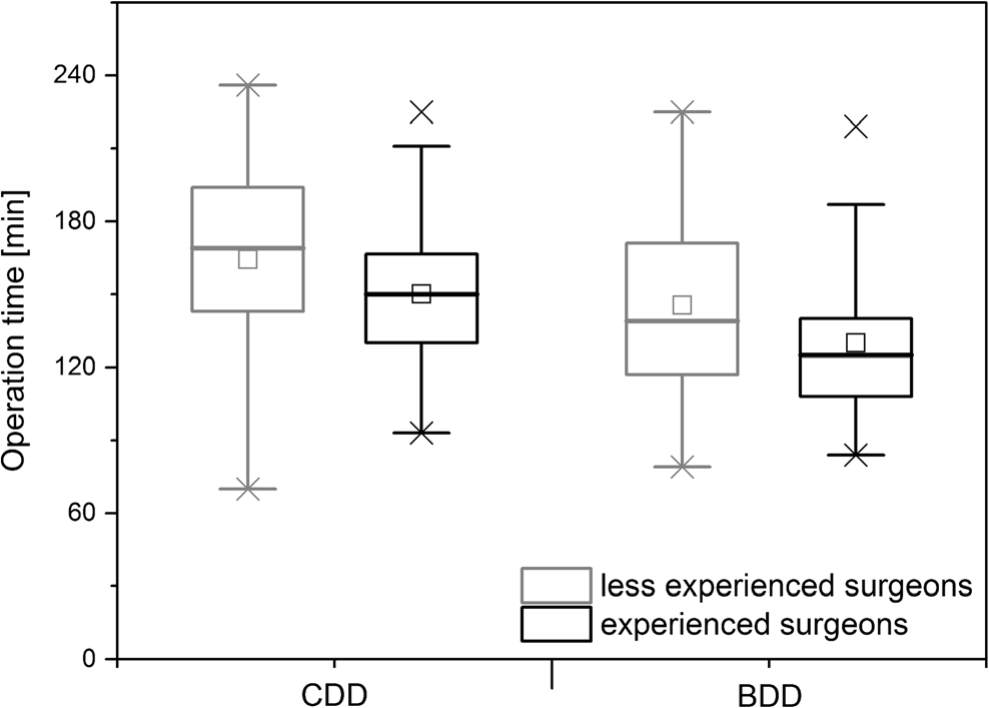
Box plots for operation time grouped by dissection device and surgeon’s experience. Boxes indicate upper and lower quartile; whiskers indicate 1.5 times the interquartile range. Crosses mark the maximum and minimum values. Significant differences for both groups between devices and overall difference between experienced surgeons and less experienced surgeons. No difference between experienced and less experienced surgeons within each device group. Significant differences are marked by asterisks

The median operation time in the CDD group at 155 min (range 70–236 min) was significantly longer than in the BDD group (127 min; range 79–225 min; Wilcoxon *W* = 5203.5, *p* << 0.001, *r* = −0.319; see also Fig. 2). Data scattering for the operation time was less pronounced in the experienced surgeon group (see whiskers in Fig. 2). For experienced surgeons, BDD shortened the operation time by a median of 25 min (CDD 150 min; BDD 125 min; *W* = 1931.5, *p* < 0.001, *r* = −0.384). For inexperienced surgeons, the operation time was reduced by 30 min (CDD 169 min; BDD 139 min; *W* = 776.5, *p* = 0.041, *r* = −0.264). A significant difference between experienced and inexperienced surgeons was observed only in the CDD group (*W* = 1932, *p* = 0.03, *r* = −0.24). For BDD, there was only noteworthy, not significant difference between experienced and less experienced surgeons (*W* = 1772, *p* = 0.057, *r* = −0.214).

**Fig. 3:**
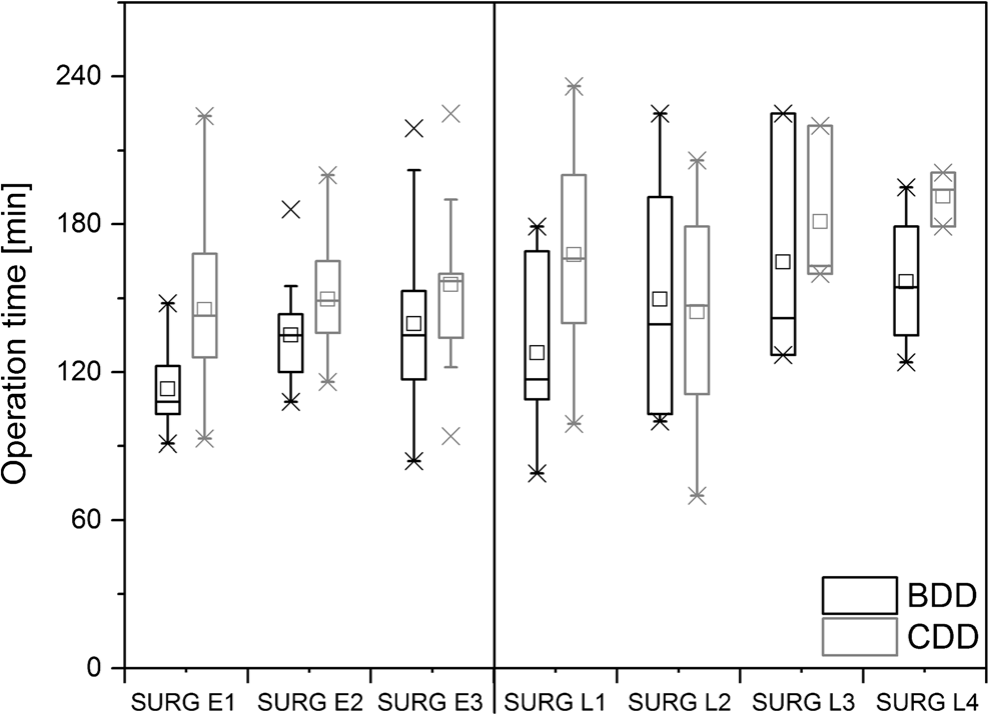
Box plots for operation time grouped by dissection device and individual surgeons. Boxes indicate upper and lower quartile; whiskers indicate 1.5 times the interquartile range. Crosses mark the maximum and minimum values. Surgeons with less experience are denoted with an L; surgeons with more experience are denoted with an E

The operation time was reduced for each individual surgeon after switching to the new device (Fig. 3). Overall, the surgeons achieved a median reduction of 22 min (8 to 49 min) when using the BDD.

### Device impact on the operation time

**Fig. 4:**
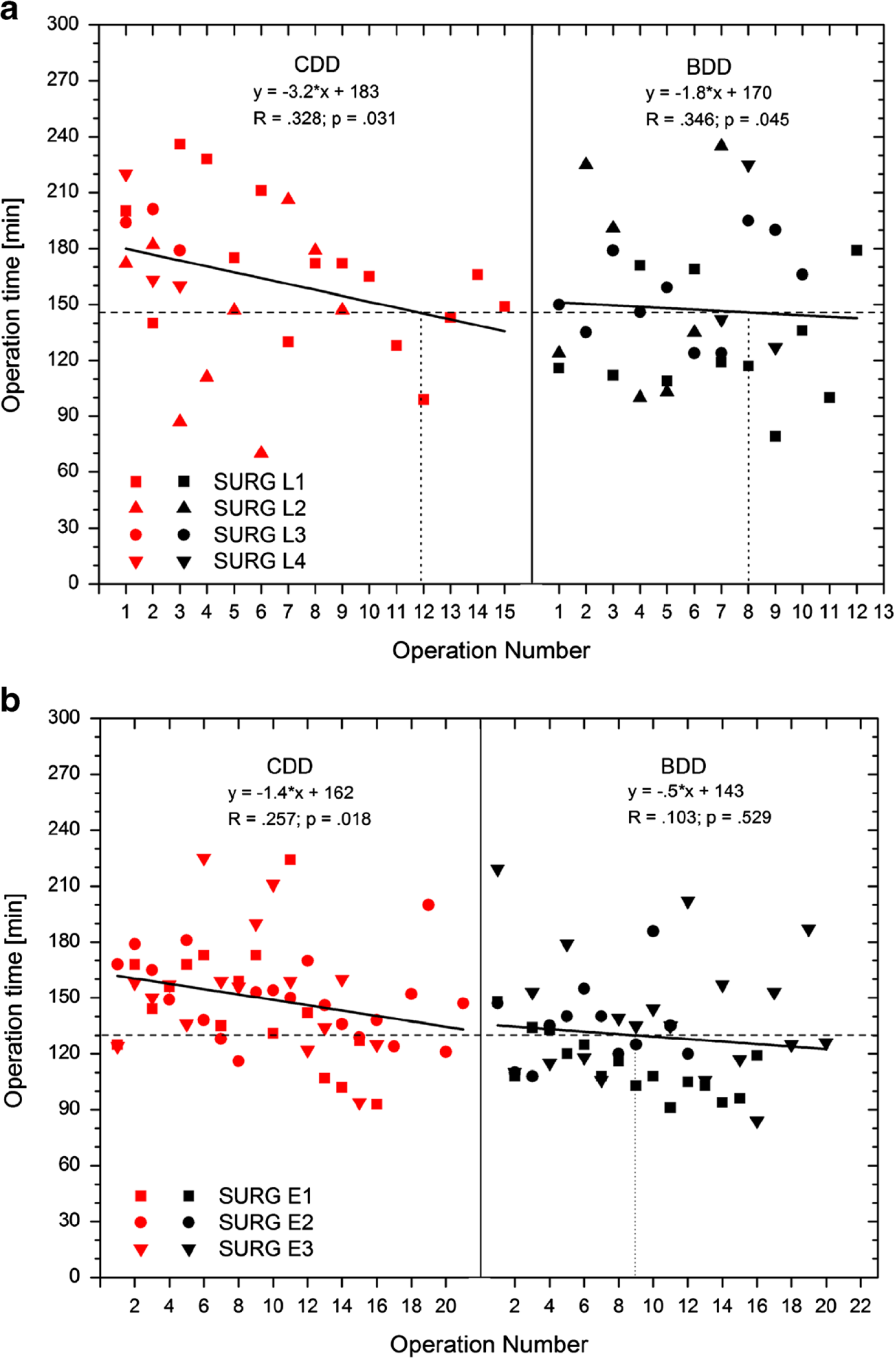
**a**, **b** Operation time with regards to the consecutive operation number of each respective surgeon. Stratification by experience: surgeons with less experience (upper panel) and surgeons with greater experience (lower panel); solid black lines indicate the linear fit estimating the correlation between operation time and number of operations performed. Dotted horizontal lines indicate the respective median value for operation time with BDD. Dotted vertical lines indicate the estimated operation number when this mean value is reached

Analysis of the operation time during the observation period revealed a reduction in the operation time independently of the selected device, thus reflecting the individual learning curve (Fig. 4). The regression lines (linear fit) were steeper in the inexperienced surgeon group (CDD slope −3.2, BDD slope −1.8; see also equations in Fig. 4a) than the regression lines in group B (experienced surgeons), and were markedly flatter for CDD (CDD −1.4, BDD −0.5; equations in Fig. 4b).^1^ As indicated in the equations in Fig. 4a, b, all regression fits were significant except for the expert surgeons with the BDD (all *p* < 0.045 except experts using BDD with *p* = 0.529). The goodness of fit was acceptable (indicated by *R* values >0.2), again except for expert surgeons using BDD. In both groups, the BDD regression line intersected the mean after between eight and nine operations (horizontal line) for the BDD operation time. For CDD, more than 12 and 20 operations, respectively, were needed to reach the mean BDD level.

**Fig. 5:**
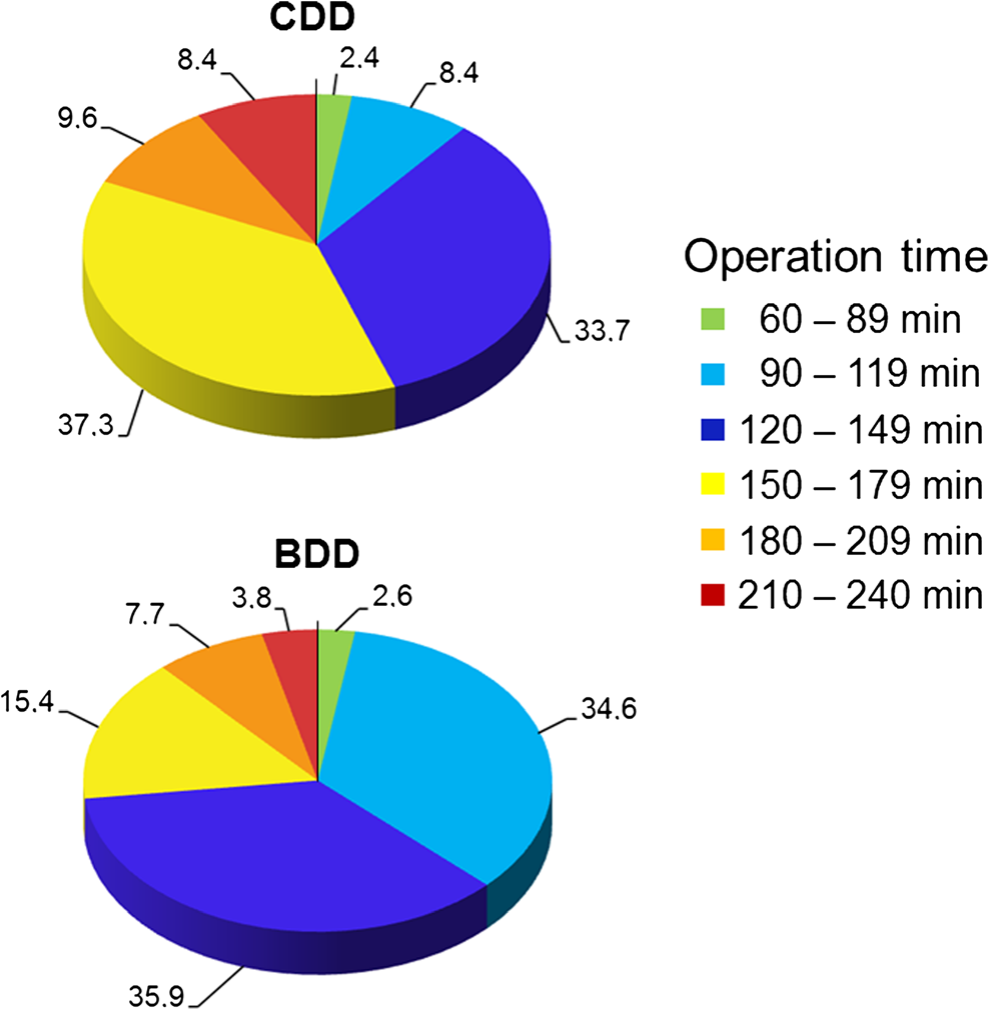
Percentage of operations in each duration group for CDD and BDD separately

Grouping of the operation time (Fig. 5) revealed that 89% of all BDD operations lasted between 90 and 180 min. If one views the time window of between 60 and 150 min, in the BDD group, 72.2% of all operations are within that window, whereas only 44.5% of procedures are within that window for the CDD operations (*p* < 0.05).

### Multifactorial analysis

A multiple linear backward stepwise regression analysis was performed to evaluate the impact of the following input variables on the operation time:

- Patient age at time of surgery
- BMI
- Type of surgery (non-oncologic vs. oncologic surgery)
- Experience of the surgeon (less experienced vs. experienced)
- Type of dissection device used (CDD vs. BDD).

A model with an good fit (*R* = 0.347, standard error of estimate = 32.8, *F* = 10.717, *p* < 0.001) and very good power of the performed test (1—*β* = 0.995, for *α* = 0.05) was found, containing only experience of the surgeon (*F* = 7.892, *p* = 0.006) and type of dissection device used (*F* = 13.866, *p* < 0.001) as predictive variables for operation time (Table 3). In the second to last step of the backward analysis, BMI was excluded from the model with *p* = 0.053, hinting at a potential effect of BMI on operation time.

**Table 3:** Results of the backward stepwise linear regression analysis

Group	Coefficient	Standard error	*F*	*p* value
Constant	164.622	4.976		
Dissection device	−19.335	5.192	13.866	*p* < 0.001
Surgeons’ experience	−15.107	5.377	7.892	*p* = 0.006

Only values for the variables predictive of surgery duration are given

## Discussion

The operation time has proved to be a suitable parameter for characterizing the learning curve [24, 25]. The operative times, calculated on the basis of the dissection and suturing time, recorded in this study are comparable with the data already published [17, 23, 29, 30]. The operation time in the present study was significantly reduced through the combination of ultrasonic scissors and bipolar coagulation mode (BDD). Even when taking the learning curve associated with laparoscopic surgical procedures into account, individual analysis of experienced and inexperienced surgeons revealed a reduction in the operation time of up to 31%, reflecting both skilled deployment of the device as well as its technical benefits. The effect was particularly conspicuous for the BDD device in the less experienced surgeon group (mean reduction of about 30-min operation time with BDD). That effect can no doubt be somewhat explained by the overall learning curve, but also by a more stringent sequence of events during surgery since BDD obviates the need for device exchange between coagulation and cutting. That hypothesis is further underpinned by the significant, but somewhat smaller, reduction in the operation time for experienced surgeons (median reduction of 25 min in the operation time). Accordingly, this appears to lend credence to the belief that it is not the learning curve alone but also the employed device which contributes as an independent factor to the reduction in the operation time.

Results of the multifactorial analysis also indicate that using a BDD instead of a CDD may have a larger impact on operation time than, for instance, BMI or type of surgery (oncological vs. non-oncological resection). However, since the overall amount of oncological resections was small, especially in the CDD group, this result should be viewed with some caution. In the period between March 2008 and February 2012, only two oncological laparoscopic sigmoid resections were performed. This is due to the fact that laparoscopic oncologic surgery was commonly performed only in recent years. It is, however, noteworthy that median operation time is shorter in the BDD group, although this group contains significantly more oncological resections, which typically take longer than non-oncological resections.

Shorter operation time is mainly interpreted in the literature as evidence of enhanced performance safety. The smooth progression of surgery in the BDD group as well as the statistically corroborated reduction in scattering of operation time data is most likely due to the fact that 72% of all operations ranged between 60 and 150 min compared with only 44% for CDD operations. Besides, correlation analysis showed that none of the patient-related factors significantly impacted the operation time. In addition, no differences were observed between BDD and CDD usage with regards to major complications such as, e.g., anastomotic leakage, which further confirms that it is safe to use BDDs despite the markedly higher heat generation in the region of the scissor shanks.

The use of the BDD appears to have had a positive effect on the learning curve, too. For comparable devices, only a flat learning curve was observed for experienced surgeons over a period of 4 years. Whereas even experienced surgeons needed *n* = 20 operations to achieve an average operation time of 145 min, that operation time was undershot already after *n* = 8 operations with BDD. That this reduction in the operation time is attributable to a learning curve effect appears unlikely; indeed, more so it would seem to be directly linked to the device. Moreover, the steep learning curve noted for inexperienced surgeons demonstrates that with greater experience, the “learning curve” effect, and accordingly the learning experience, declines. That observation can, however, only be validated in a prospective randomized setting.

The retrospective study design chosen here was chosen to record over a long observation period all the influence factors at play in routine clinical care. The inherent advantage of this study design is, in addition to an observation period spanning several years, the avoidance of study bias arising from patient selection as practiced in prospective randomized trial settings. However, this study design suffers the drawback of affording only limited insights into causal relationships, and has little scope to identify confounders. On the other hand, this empirical evidence demonstrates that, regardless of experience, learning curve, and individual patient-specific factors, medical technical innovations can positively impact both the learning curve and the operation time.

## Conclusion

In summary, the combination of ultrasonic cutting technology and simultaneous cauterization with a BDD results in significantly faster operation time for both trainee and experienced surgeons. The learning curve appears to be steeper on using BDD which might be indicative of enhanced performance safety.

## Footnotes

1 For instance, a slope of −3.2 indicates an average decrease in surgery duration of 3.2 min per conducted surgery.

## References

[1] Nolan GJ, Howell S, Hewett P (2015). Impact of three-dimensional imaging in acquisition of laparoscopic skills in novice operators. J Laparoendosc Adv Surg Tech.

[2] Reiser S, Kohn N, Schneider A, Feußner H, Wilhelm D (2015). 3D-Visualisierung in der interventionellen Medizin. Endoskopie Heute.

[3] Fagotti A, Vizzielli G, Fanfani F, Gallotta V, Rossitto C, Costantini B (2014). Randomized study comparing use of THUNDERBEAT technology vs standard electrosurgery during laparoscopic radical hysterectomy and pelvic lymphadenectomy for gynecologic cancer. J Minim Invasive Gynecol.

[4] Eto K, Omura N, Haruki K, Uno Y, Ohkuma M, Nakajima S (2015). A comparison of laparoscopic energy devices on charges in thermal power after application to porcine mesentery. Surg Laparosc Endosc Percutan Tech.

[5] Sutton P, Awad S, Perkins A, Lobo D (2010). Comparison of lateral thermal spread using monopolar and bipolar diathermy, the Harmonic Scalpel^™^ and the Ligasure^™^. Br J Surg.

[6] Seehofer D, Mogl M, Boas-Knoop S, Unger J, Schirmeier A, Chopra S (2012). Safety and efficacy of new integrated bipolar and ultrasonic scissors compared to conventional laparoscopic 5-mm sealing and cutting instruments. Surg Endosc.

[7] Milsom J, Trencheva K, Monette S, Pavoor R, Shukla P, Ma J (2012). Evaluation of the safety, efficacy, and versatility of a new surgical energy device (THUNDERBEAT) in comparison with Harmonic ACE, LigaSure V, and EnSeal devices in a porcine model. J Laparoendosc Adv Surg Tech A.

[8] Rassweiler JJ, Teber D (2016). Advances in laparoscopic surgery in urology. Nature reviews. Urology.

[9] Lin HZ, Ng Y, Agarwal A, Fong Y (2013) Application of a new integrated bipolar and ultrasonic energy device in laparoscopic hysterectomies. ISRN Minim Invasiv Surg. 10.1155/2013/453581

[10] Velayutham V, Fuks D, Nomi T, Kawaguchi Y, Gayet B (2015) 3D visualization reduces operating time when compared to high-definition 2D in laparoscopic liver resection: a case-matched study. Surg Endosc 30(1):147–153 10.1007/s00464-015-4174-1 25805241

[11] Jacobs M, Verdeja J, Goldstein H (1991). Minimally invasive colon resection (laparoscopic colectomy). Surg Laparosc Endosc Percutan Tech.

[12] Köckerling F, Schneider C, Reymond MA, Scheidbach H, Scheuerlein H, Konradt J (1999). Laparoscopic resection of sigmoid diverticulitis. Results of a multicenter study. Laparoscopic colorectal surgery study group. Surg Endosc.

[13] Köhler L, Sauerland S, Neugebauer E, Caprilli R, Fingerhut A, Haboubi N (1999). Diagnosis and treatment of diverticular disease. Surg Endosc.

[14] Wong WD, Wexner SD, Lowry A, Vernava A, Burnstein M, Denstman F (2000). Practice parameters for the treatment of sigmoid diverticulitis—supporting documentation. Dis Colon Rectum.

[15] Rafferty J, Shellito P, Hyman NH, Buie WD (2006). Practice parameters for sigmoid diverticulitis. Dis Colon Rectum.

[16] Leifeld L, Germer C, Böhm S, Dumoulin F, Häuser W, Kreis M (2014). S2k Leitlinie Divertikelkrankheit/Divertikulitis. Z Gastroenterol.

[17] Gervaz P, Inan I, Perneger T, Schiffer E, Morel P (2010). A prospective, randomized, single-blind comparison of laparoscopic versus open sigmoid colectomy for diverticulitis. Ann Surg.

[18] Lacy AM, Delgado S, Castells A, Prins HA, Arroyo V, Ibarzabal A (2008). The long-term results of a randomized clinical trial of laparoscopy-assisted versus open surgery for colon cancer. Ann Surg.

[19] Scheidbach H, Schneider C, Hügel O, Scheuerlein H, Bärlehner E, Konradt J (2003). Oncological quality and preliminary long-term results in laparoscopic colorectal surgery. Surg Endosc.

[20] Franklin ME, Rosenthal D, Abrego-Medina D, Dorman JP, Glass JL, Norem R (1996). Prospective comparison of open vs. laparoscopic colon surgery for carcinoma. Dis Colon Rectum.

[21] Franklin ME, Kazantsev GB, Abrego D, Diaz-e JA, Balli J, Glass JL (2000). Laparoscopic surgery for stage III colon cancer. Surg Endosc.

[22] Schneider C, Scheidbach H, Scheuerlein H, Köckerling F (2000). Prospective multicenter study of laparoscopic colorectal surgery. Quality assurance during introduction of new methods. Zentralbl Chir.

[23] Klarenbeek BR, Veenhof AA, Bergamaschi R, van der Peet DL, van den Broek WT, de Lange ES (2009). Laparoscopic sigmoid resection for diverticulitis decreases major morbidity rates: a randomized control trial: short-term results of the Sigma Trial. Ann Surg.

[24] Simons AJ, Anthone GJ, Ortega AE, Franklin M, Fleshman J, Geis WP (1995). Laparoscopic-assisted colectomy learning curve. Dis Colon Rectum.

[25] Schlachta CM, Mamazza J, Seshadri PA, Cadeddu M, Gregoire R, Poulin EC (2001). Defining a learning curve for laparoscopic colorectal resections. Dis Colon Rectum.

[26] Dinçler S, Koller MT, Steurer J, Bachmann LM, Christen D, Buchmann P (2003). Multidimensional analysis of learning curves in laparoscopic sigmoid resection. Dis Colon Rectum.

[27] Tekkis PP, Senagore AJ, Delaney CP, Fazio VW (2005). Evaluation of the learning curve in laparoscopic colorectal surgery: comparison of right-sided and left-sided resections. Ann Surg.

[28] Park J-S, Kang S-B, Kim S-W, Cheon G-N (2007). Economics and the laparoscopic surgery learning curve: comparison with open surgery for rectosigmoid cancer. World J Surg.

[29] Ritz J, Reissfelder C, Holmer C, Buhr H (2008). Results of sigma resection in acute complicated diverticulitis: method and time of surgical intervention. Chirurg.

[30] Jones OM, Stevenson AR, Clark D, Stitz RW, Lumley JW (2008). Laparoscopic resection for diverticular disease: follow-up of 500 consecutive patients. Ann Surg.

